# Microvascular density and hypoxia-inducible factor in intraepithelial vocal fold lesions

**DOI:** 10.1007/s00405-019-05355-2

**Published:** 2019-03-06

**Authors:** Anna Rzepakowska, Michał Żurek, Jakub Grzybowski, Paweł Pihowicz, Barbara Górnicka, Kazimierz Niemczyk, Ewa Osuch-Wójcikiewicz

**Affiliations:** 1 Otolaryngology Department, Medical University of Warsaw, Warsaw, Poland; 2 Students Scientific Research Group by Otolaryngology Department, Medical University of Warsaw, Warsaw, Poland; 3 Department of Pathology, Medical University of Warsaw, 7, Pawińskiego Str., 02-004 Warsaw, Poland

**Keywords:** Intraepithelial lesions, Dysplasia, Glottic cancer, Larynx, Microvascular density, Hypoxia, Angiogenesis

## Abstract

**Objective:**

The promotion of neovascularisation is a crucial aspect of carcinogenesis. The study evaluates the microvascular density (MVD) and expression of hypoxia-induced factor (HIF-1α) in hypertrophic vocal fold (VF) lesions of different histopathological states including non-dysplastic, low-grade, high-grade dysplasia and invasive glottic cancer.

**Materials and methods:**

Histological specimens collected from patients diagnosed and treated in a single centre with different histological grades were immunohistochemically stained with CD31, CD34 and HIF-1α. Of the total number of 77 analysed VF specimens, 20 were non-dysplastic, 20 had low-grade dysplasia, 17 high-grade dysplasia and 20 were invasive cancers.

**Results:**

The highest mean value for MVD evaluated with expression of CD31 (MVD CD31) was 21.23 ± 14.46 and identified in the low-grade dysplasia group. The average MVD CD31 was 13.74 ± 5.56 and 20.11 ± 9.28 in the high-grade dysplasia and invasive cancer group, respectively. The highest MVD evaluated with CD34 (MVD CD34) was revealed for invasive cancer 35.64 ± 17.21. The MVD CD34 was higher for low-grade than in high-grade dysplasia (25.87 ± 12.30 vs 24.65 ± 15.92, respectively). The expression of HIF-1α was strong or very strong in 60% of non-dysplastic lesions, 100% of low-grade dysplasia cases, 53% of high-grade dysplasia cases and 50% of invasive cancer cases. The comparison of MVD CD31 with MVD CD34 revealed a strong positive correlation (*ρ* value 0.727). The comparison of both MVD CD31 and MVD CD34 with HIF-1α resulted in no linear relationship (*ρ* value of 0.143 and 0.165, respectively).

**Conclusion:**

The stage of low-grade dysplasia in intraepithelial vocal fold lesions is related to significant advancement of angiogenesis together with the highest hypoxia level.

**Electronic supplementary material:**

The online version of this article (10.1007/s00405-019-05355-2) contains supplementary material, which is available to authorized users.

## Introduction

The model of the genetic changes responsible for progression of hypertrophic laryngeal lesions to dysplastic and invasive cancer is still incomplete and lacking [1]. The architectural and cytological disorder found within epithelial cells can be histopathologically classified as squamous hyperplasia, dysplasia or invasion. This pathology is controlled by a multistep process of genetic changes, which not only influence the potential for cellular multiplication at this level, but also inevitably alter the surrounding microenvironment indirectly, providing for the most optimal conditions for tumour expansion [2]. Neoangiogenesis, defined as the growth of new vessels, is essential for neoplastic lesions during their process of progression and is, therefore, one of the fundamental events in the carcinogenesis [3]. The formation of neoplastic microvessels is stimulated by several other growth factors including vascular endothelial growth factor (VEGF), transforming growth factor (TGF) and various cytokines secreted by the altered cells [3]. However other triggering conditions of the microenvironment, including the state of hypoxia, are also critical for these processes of tumour formation [4].

Hypertrophy of vocal fold (VF) epithelium is most often caused by smoking, particularly when combined with the consumption and abuse of high-percentage alcohol. Active gastroesophageal reflux disease (GERD) in patients is another risk factor for the emerging hypertrophy. Exposure to the mentioned agents is responsible for promotion of both non-dysplastic and dysplastic lesions, as well as invasive cancer [1]. Despite there being new and emerging literature on human papilloma virus (HPV) as related to neoplasia within the larynx, the published data remain controversial and necessitate further investigation [5]. The clinically identified hyperplasia or leukoplakia of the vocal folds can provide for a wide range of histopathological diagnoses. During the last few decades, there was no consensus on a unified pathological classification of precancerous laryngeal lesions and three different rating systems were valid and in use [5]. Fortunately, the newest 4th edition of the World Health Organization Classification of Head and Neck Tumours from 2017 was able to consolidate the terminology and grading systems into a more cohesive and uniform framework. Currently under this novel classification system, precancerous lesions are classified according to their grade into either low- or high-grade dysplasia, or invasive cancer [5]. Carcinoma in situ can be distinguished from high-grade dysplasia, through the presence of severe nuclear and cytologic atypia, but is only done so if it influences the treatment decision [5]. Morphologically, the criteria for low-grade dysplasia include pathologies that range from squamous hyperplasia to basal cell enlargement, display no or minimal cellular and nuclear atypia of the epithelium, and is limited to the lower one-half of its thickness. High-grade dysplasia, on the other hand, is diagnosed when atypical epithelial cells are present and involve the lower one-half of the epithelium, with the potential to reach full thickness. Infiltration of atypical cells through the basal membrane is the definition of invasive cancer [5].

VF epithelium is a layer composed of stratified squamous cells, where only the deepest layer of basal cells has contact with the stromal tissue. In light of current research, the previous theory that the epithelial surface becomes deprived of blood vessels has been undermined [4]. The microarchitecture of stromal blood vessels provides numerous sprouts that penetrate with a thin layer of stromal tissue upwards between epithelial cells [4]. A study by Arens et al. confirmed the thickness of the epithelial layer increases in accordance with the grade of premalignant VF lesions [6]. Such epithelial proliferation translates into an increased demand for nutrients. Neoangiogenesis is, therefore, inevitably preceded by a level of ischemia [4]. Hypoxia not only promotes vascular growth, but also favours the synthesis of procarcinogenic factors, including hypoxia-inducible factor (HIF-1α), which as a consequence may initiate malignant transformations [7].

Our study was designed to investigate the relation of microvascular density and hypoxia in premalignant and malignant vocal fold lesions and enable for a better understanding of the mechanisms involved in the carcinogenesis of the laryngeal epithelium. This can be further utilised in the future development of more targeted and individualized therapies, particularly during the course of early treatment and prevention, where changes to intervention can influence patient outcomes.

## Materials and methods

The protocol of the study was approved by the Institutional Ethic Review Board of the local Medical University.

This retrospective study was performed in 2018 by searching archived medical records and paraffin-embedded tissue blocks of patients treated with laryngeal microsurgery in our department from 2017 to 2018 due to suspicious hypertrophic lesions of the VFs. The design of the study assumed inclusion of 80 patients with different histopathological (HP) grades of VF lesions: non-dysplastic (stromal edema, inflammatory changes), low-grade dysplasia, high-grade dysplasia (with carcinoma in situ) and invasive cancer. The exclusion criteria included a history of radiotherapy to the neck area, papillomatosis related to HPV infection, and a history of immunodeficiency or prior systemic chemotherapy. A total of 79 patients (19 in the high-grade dysplasia group and 20 cases for each of the remaining three groups) were collected. The laryngeal specimens obtained during microsurgical resection or tissue biopsy were re-examined by two histopathologists, blinded to the previous diagnosis, to confirm the histopathology result. In one case of high-grade dysplasia, there was uncertainty regarding the invasion into the basal membrane since it could not be resolved due to the vestigial stroma as this was thermally damaged with the laser during resection. Another case of high-grade dysplasia showed findings suspicious of a coexisting HPV infection and was, therefore, rejected. Ultimately, a total number of 77 cases with a confirmed histopathological diagnosis were included for further analysis.

### Immunohistochemistry

Four serial sections of 3–5 µm thickness were obtained from 77 formalin-fixed paraffin-embedded tissue blocks with a microtome (HM 340E Electronic Rotary Microtome, Thermo Shandon). All sections were stained with hematoxylin and eosin (H&E) in an automatic tissue processor (ASP 6026, Leica, USA). Corresponding sections were stained with anti-CD31 antibody (clone: JC70A; DAKO/Agilent, USA), anti-CD34 antibody (clone: QBEnd10; DAKO/Agilent, USA) and anti-HIF-1α antibody (clone: EP1215Y; Abcam, UK).

Immunostaining was performed using EnVision FLEX, High pH (Link) (DAKO/Agilent, USA) and Autostainer Link 48 (DAKO/Agilent, USA).

In brief, the tissue sections were deparaffinized by incubation in a wash buffer, pH 9.0, at 98 °C for 20 min in PT Link station (DAKO/Agilent, USA). After 10 min of incubation in the wash buffer at room temperature, the endogenous peroxidase activity was blocked through immersion in a 3% hydrogen peroxide solution for 10 min. The slides were then incubated for 20 min with the primary antibodies, followed by the addition of the secondary antibody. In the case of anti-CD31 antibody, the reaction was strengthened through the addition of Mouse LINKER according to the producer’s recommendations. Visualization was achieved by the application of 3,3′-diaminobenzidine. Finally, cell nuclei were counterstained with hematoxylin. The tissue sections were dehydrated with graded concentrations of ethanol, cleared with xylene and covered with tape cover slippers (Klinipath, The Netherlands).

The assessment of the immunostainings was carried out by two examiners using light microscopy and the results were averaged.

### Evaluation of microvessel density

**Fig. 1:**
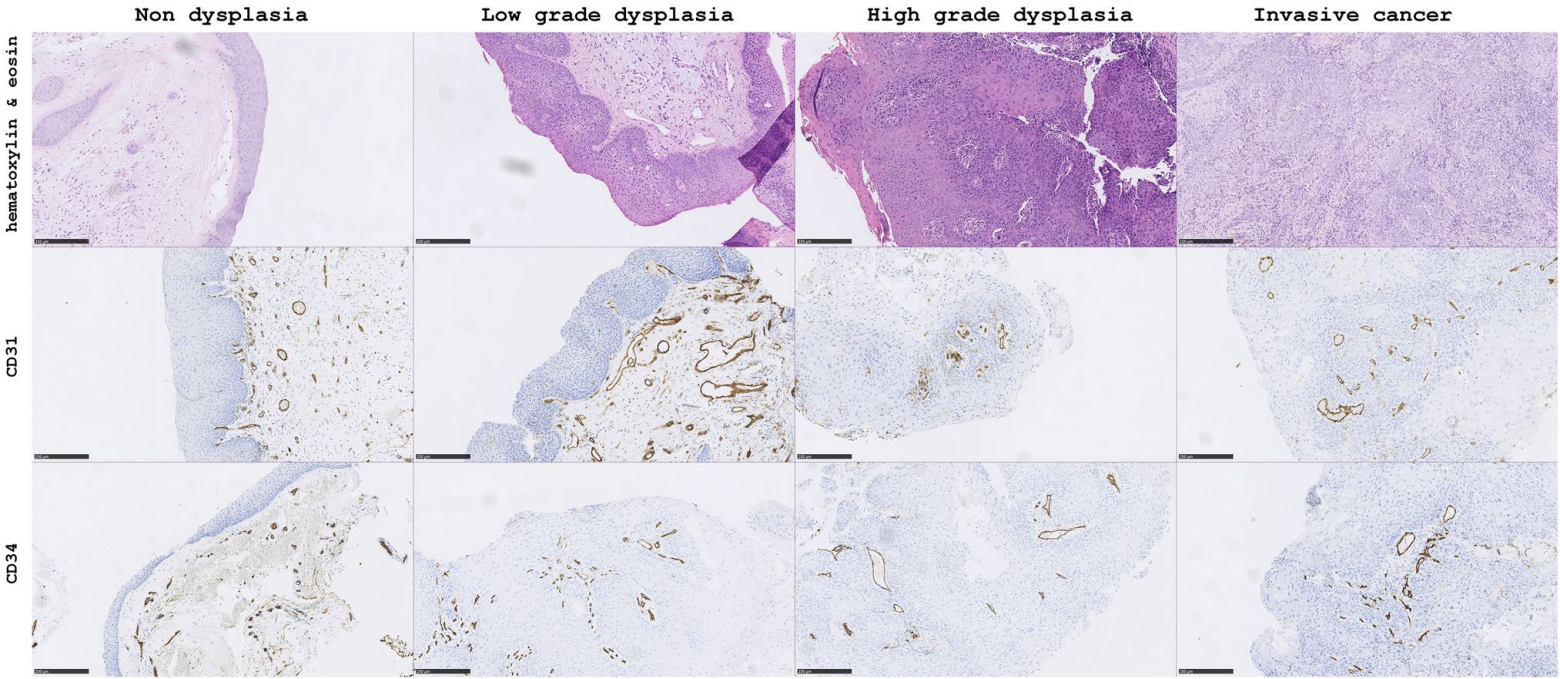
The results of hematoxylin and eosin staining, CD31 and CD34 immunostaining for non-dysplastic, low-grade dysplasia, high-grade dysplasia and invasive cancer intraepithelial vocal fold lesions at × 100 magnification

First, each slide was examined at low magnification (40 ×), selecting regions with the highest density of vessels (hot spots) before being viewed at high magnification (200 ×). CD31-positive or CD34-positive vessels were counted in three or five hot spots, respectively. Only continuous, membranous staining was considered as positive. Any large microvessel with a lumen or any single, separated endothelial cell was given a count of one. The vessels were counted within the epithelium and at the epithelium/stroma edge. Figure 1 presents examples of H&E and immunostaining for both CD31 and CD34 in each grade of intraepithelial VF lesions at 100 × magnification.

### Evaluation of HIF-1α expression

**Fig. 2:**
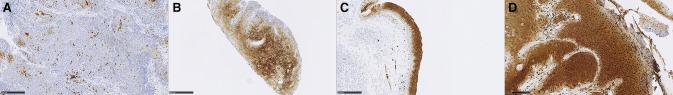
The categories of expression of HIF-1α immunostaining in intraepithelial vocal fold lesions at × 100 magnification: **a** 1–25% positive nuclei—weakly positive expression, **b** 26–50% positive nuclei—moderately positive expression, **c** 51–80% positive nuclei—strong positive expression, **d** more than 80% positive nuclei—very strong positive expression

There are no standardized levels of HIF-1α staining. The evaluation of this staining was only quantitative and a specimen was considered as positive if more than 1% of cell nuclei were stained. Membranous or cytoplasmic staining was not assessed in this study. Using the methodology found in majority of publications with positively stained samples as a basis, the following classification was adopted: 1–25% positive nuclei—weakly positive expression, 26–50% positive nuclei—moderately positive expression, 51–80% positive nuclei—strong positive expression, more than 80% positive nuclei—very strong positive expression. Figure 2 presents four categories of expression of HIF-1α immunostaining in intraepithelial VF lesions at 100 × magnification.

### Statistical analysis

All parameters were evaluated using SPSS 18.0 and Statistica 13 for the analyses and a *p* value lower than 0.05 was considered statistically significant. The differences between groups with different histopathological diagnosis and expression of CD31, CD34 and HIF-1α were compared using nonparametric Kruskal–Wallis and Mann–Whitney *U* tests. Box plots were used for a graphical representation of the received results. The Spearman rank correlation coefficients were used to calculate the correlation between microvessel density (MVD) and the expression of CD31 and CD34 alike, as well as the relationship between MVD and HIF-1α levels.

## Results

The immunohistochemical analysis was finally performed on sections derived from 77 VF lesions. The patient age ranged from 23 to 89 years, with a median of 61.96 years and standard deviation of 11.62 years. There were 54 males and 23 females among the studied samples. The groups with non-dysplastic lesions, low-grade dysplasia and invasive cancer consisted of 20 patients each. The group with high-grade dysplasia included 17 patients. Detailed demographic data of each group are presented in Table 1.

**Table 1:** Characteristic of patients with vocal fold lesions

	Number	Female/male	Mean age ± SD; range (years)
Non-dysplasia	20	12/8	55.15 ± 12.38 (range 23–71)
Low-grade dysplasia	20	3/17	61.55 ± 10.65 (range 35–85)
High-grade dysplasia	17	4/13	64.94 ± 11.25 (range 37–89)
Invasive cancer	20	4/16	66.65 ± 9.31 (range 56–88)
All patients	77	23/54	61.96 ± 11.62 (range 23–89)

**Fig. 3:**
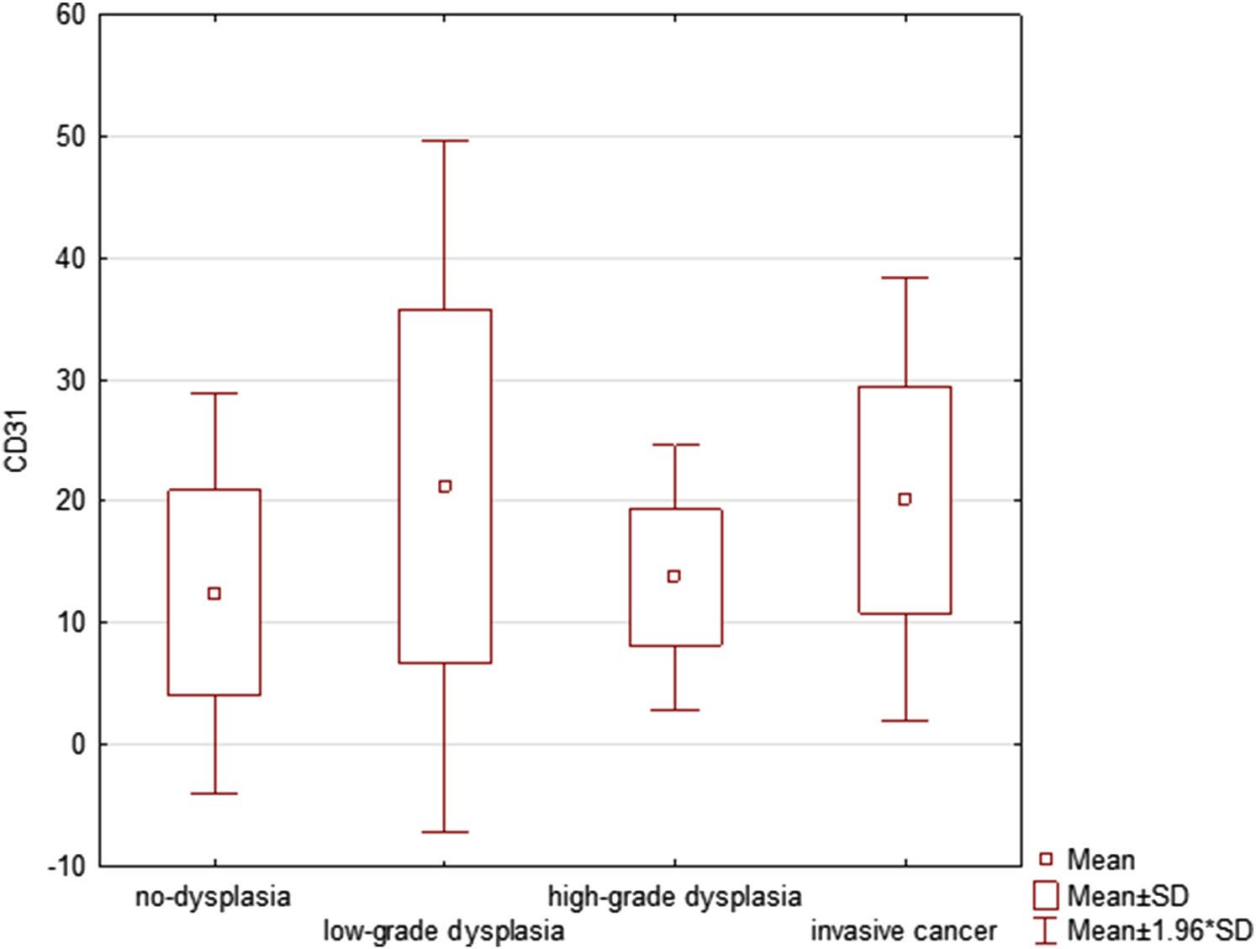
The boxplot diagram presenting microvascular density with CD31 (mean, standard deviation) for non-dysplastic, low-grade dysplasia, high-grade dysplasia and invasive cancer of vocal folds

Microvascular density evaluated with expression of CD31 (MVD CD31) in endothelial cells revealed statistically significant differences (*p* = 0.0115) in microvascular count between the analysed groups. The highest mean value of MVD CD31 expression (21.23 ± 14.46) was identified in lesions with low-grade dysplasia. Interestingly, the average MVD CD31 was 13.74 ± 5.56 for the high-grade dysplasia group and 20.11 ± 9.28 for the invasive cancer group. Detailed statistics of MVD CD31 expression for each group are presented in Table 2. Figure 3 presents a graphical representation of data for MVD CD31 expression in the analysed groups.

**Table 2:** The evaluated microvessel density (MVD) with CD31 in different grades of histopathological diagnosis

	CD31 No. of specimens	CD31 MVD Mean	CD31 MVD 95% confidence interval (CI)	CD31 MVD Standard deviation (± SD)	CD31 MVD Standard error	CD31 MVD Range	*p* value
Non-dysplasia	20	12.47	8.53–16.40	8.41	1.88	3.00–33.33	0.02^#/^*
Low-grade dysplasia	20	21.23	14.47–28.00	14.46	3.23	5.33–56.00	
							0.12^#^
High-grade dysplasia	17	13.74	10.89–16.60	5.56	1.35	5.67–23.67	
							0.03^#/^*
Invasive cancer	20	20.11	15.77–24.46	9.28	2.07	6.00–45.33	
All patients	77	17.01	14.60–19.43	10.63	1.21	3.00–56.00	0.01^†/^*

^#^*p* value for the Mann–Whitney test for the comparison of values between the two groups

^†^*p* value for Kruskal–Wallis for overall assessment of differences between groups

*Statistically significant *p* value (< 0.05)

**Fig. 4:**
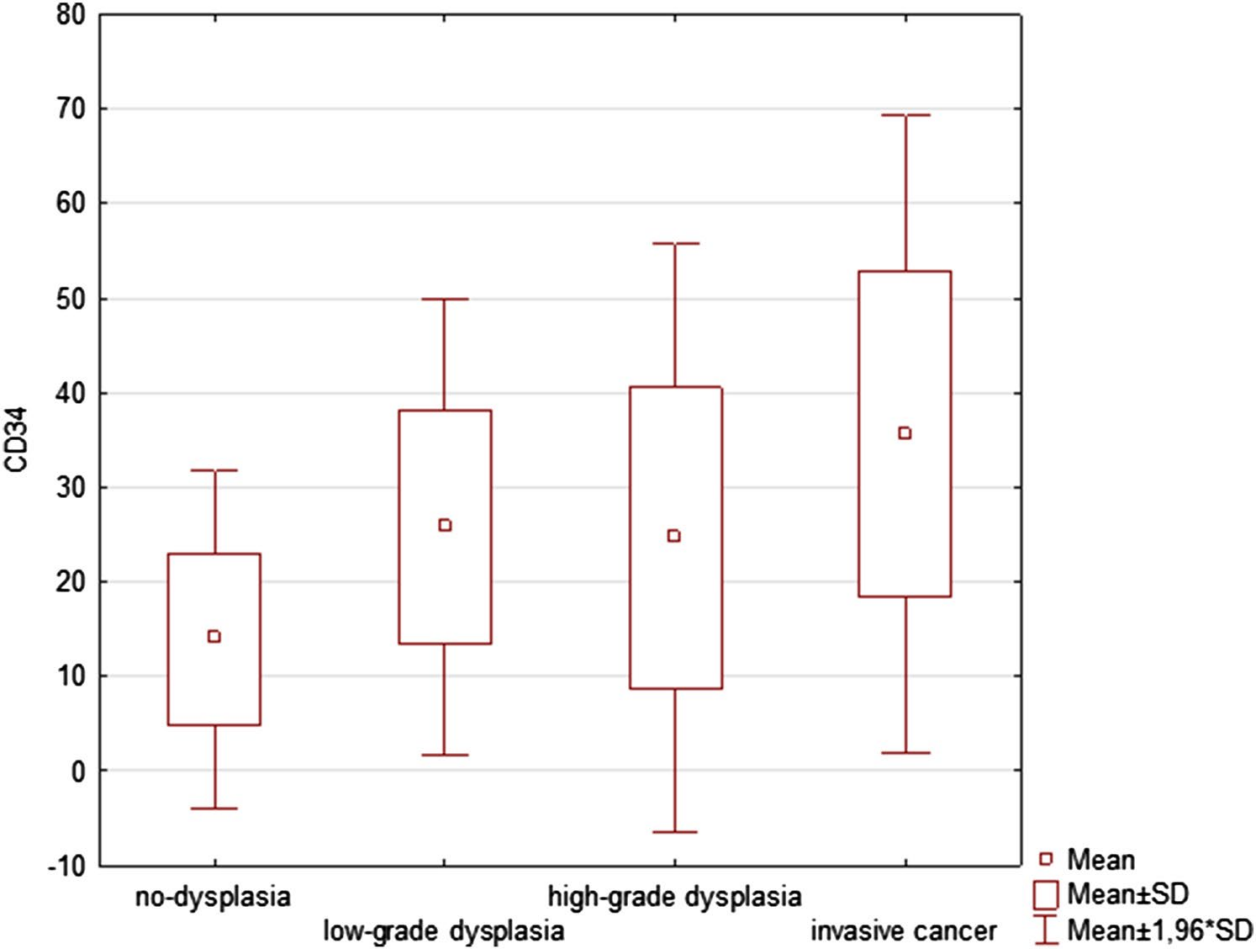
The box plot diagram presenting microvascular density with CD34 (mean, standard deviation) for non-dysplastic, low-grade dysplasia, high-grade dysplasia and invasive cancer of vocal folds

CD34 expression in endothelial cells was used for the comparative assessment of microvascular density (MVD CD34). Statistical analysis confirmed a significant difference (*p* = 0.0001) in microvascular count between the analysed groups for this method as well. However, the highest mean value for MVD CD34 was revealed this time in lesions with invasive cancer 35.64 ± 17.21, but was also found to be higher in low-grade than in high-grade dysplasia (25.87 ± 12.3 vs 24.65 ± 15.92, respectively). The statistical results of MVD CD34 are presented in Table 3. Figure 4 presents the statistical data graphically for MVD CD34 in the analysed groups.

**Table 3:** The evaluated microvessel density (MVD) with CD34 in different grades of histopathological diagnosis

	CD34 MVD No. of specimens	CD34 MVD Mean	CD34 MVD 95% confidence interval (CI)	CD34 MVD Standard deviation (± SD)	CD34 MVD Standard error	CD34 MVD Range	*p* value
Non-dysplasia	20	13.96	9.69–18.23	9.12	2.04	2.00–31.80	0.004^#/^*
Low-grade dysplasia	20	25.87	20.11–31.63	12.30	2.75	2.80–49.40	
							0.61^#^
High-grade dysplasia	17	24.65	16.46–32.83	15.92	3.86	4.00–57.00	
							0.03^#/^*
Invasive cancer	20	35.64	27.59–43.69	17.21	3.85	10.00–92.60	
All patients	77	25.04	21.47–28.62	15.75	1.80	2.00–96.60	0.0001^†/^*

^#^*p* value for the Mann–Whitney test for the comparison of values between the two groups

^†^*p* value for Kruskal–Wallis for overall assessment of differences between groups

*Statistically significant *p* value (< 0.05)

On immunohistochemical staining of laryngeal specimens for HIF-1α, strong or very strong expression was identified in 60% of non-dysplastic lesions, 100% of low-grade dysplasia, 53% of high-grade dysplasia and 50% of invasive cancers. The details concerning expression of HIF-1α in different grades of histopathological diagnoses are presented in Table 4.

**Table 4:** The evaluated expression of hypoxia-inducible factor (HIF-1α) in different grades of histopathological diagnosis

	HIF 1α number of specimens	Weakly positive expression (HIF 1α reaction in 1–10% of nuclei) (%)	Moderately positive expression (HIF 1α reaction in 11–50% of nuclei) (%)	Strong positive expression (HIF 1α reaction in 51–80% of nuclei) (%)	Very strong positive expression (HIF 1α reaction in > 80% of nuclei) (%)	*p* value
Non-dysplasia	20	1 (5)	7 (35)	8 (40)	4 (20)	< 0.0001^#/^*
Low-grade dysplasia	20	0	0	2 (10)	18 (90)	
						0.0001^#/^*
High-grade dysplasia	17	2 (11.7)	6 (35.3)	5 (29.4)	4 (23.5)	
						0.96^#^
Invasive cancer	20	3 (15)	7 (35)	4 (20)	6 (30)	
All patients	77	6 (7.8)	20 (25.9)	19 (24.6)	32(41.5)	< 0.0001^†/^*

^#^*p* value for the Mann–Whitney test for the comparison of values between the two groups

^†^*p* value for Kruskal–Wallis for overall assessment of differences between groups

*Statistically significant *p* value (< 0.05)

The comparison of MVD evaluated with CD31 and CD34 revealed a strong positive correlation of both methods (*ρ* value of 0.727; range 1 to − 1). However, individual comparisons of both MVD CD31 and MVD CD34 with HIF-1α resulted in no linear relationship (*ρ* values of 0.143 and 0.165, respectively).

## Discussion

Laryngeal lesions, of premalignant and malignant nature, have been a topic of discussion and debate focusing on laryngeal pathology. It has been proven that 90% of laryngeal cancers are preceded by precancerous lesions of leukoplakia or mucosa hypertrophy characterized histopathologically as epithelial dysplasia [7]. Therefore, identification and management of intraepithelial lesions are imperative in the prevention of invasive neoplasia. Understanding the mechanisms of the processes involved in malignant transformation forms the basis of proper treatment. The period from first diagnosis of laryngeal dysplasia to malignant transformation correlates with the grade of dysplasia [8]. Identification of biochemical factors that correlate with the progression of intraepithelial dysplasia progression to invasive cancer would enable for individualisation of treatment strategies in addition to early intervention. Current therapy for non-dysplastic and low-grade dysplasia includes two options: “watchful waiting” or microsurgical excision. For high-grade dysplasia, a more radical excision is proposed (subepithelial cordectomy of type II). Present day recommendations for invasive early glottic cancer include surgical excision or radiotherapy as equivalent methods. Publications concerning angiogenesis or hypoxia in intraepithelial laryngeal lesions are very scarce, however. Kojc et al. found that in contrast to invasive cancer, laryngeal intraepithelial lesions stained positively for CD34 stromal cells but there was no reaction with myofibroblast antibodies in the stroma [9]. Szafranowski et al. reported a significant increase in microvessel density identified with CD34 and CD105 in laryngeal cancer and dysplasia when compared to the healthy control group [10]. Prior research on microvessel density in intraepithelial lesions of the oesophagus and cervix confirmed an increased vessel number associated with the transformation from intraepithelial lesions to invasive cancers [11, 12]. CD31 and CD34 are most commonly utilised as endothelial markers in the literature. While CD31 is detected in matured endothelial cells, CD34 is expressed in both precursor and differentiated endothelial cells, making this marker less specific. Anti-CD34 antibodies also immunostain lymphatic endothelial cells, fibroblasts and adipocytes, and may falsely elevate the values for CD34 MVD in comparison to the CD31 MVD count.

Studies centred on angiogenesis and vessel formation most often correlate MVD with inflammatory states, notably regarding the number of macrophages. We did not find any works comparing hypoxia with MVD in intraepithelial lesions. During the study design, it was expected to observe an increase in MVD with the rise in severity of histopathological grade in laryngeal lesions. An increase in hypoxemia factor seemed to correlate with increased neovascularization. It is, however, noteworthy that mean vessel number was higher in the low-grade as opposed to high-grade dysplasia, as was witnessed in both methods of MVD evaluation. Even more intriguing is the pattern of HIF-1α expression in low-grade dysplasia, where 100% of lesions had a positive reaction in more than 51% of cell nuclei found within lesions. Tobacco exposure and low-pH states chronically irritate the epithelium, putting it under much oxidative stress. The pathological analysis of epithelial thickness in different stages of intraepithelial lesions performed by Arens et al. confirmed the epithelium becomes thicker with the progression of HP status [6]. This strong expression of HIF-1α during the early stage of dysplasia can be associated with an increase in nutritional demands of proliferating cells. This elevated hypoxia factor may not only promote further neoangiogenesis, but also influence transcription at this stage and development of cellular atypia and invasiveness.

Using prior research on angiogenesis in precancerous lesions and laryngeal cancer as a basis, the analysis of MVD and hypoxia may result in treatment individualisation in the future. However, a consensus about the influence of vascular density on laryngeal cancer prognosis has yet to be reached. A high expression of CD31 and VEGF was affirmed by recent studies to correlate with poorer clinical outcomes, including reoccurrence and survival rates, especially concerning laryngeal cancer at the early stage [13–15]. Pignatoro et al. suggested that angiogenesis may be the crucial aspect in early stages of laryngeal carcinogenesis [15]. Kwon et al. reported on enhanced expression of HIF-1α in early laryngeal cancer and its association with radio-resistance [16]. It was suggested for patients with early-stage laryngeal carcinomas displaying a high expression of HIF-1α, treatment modalities alternative to primary radiotherapy should be considered in these cases [16].

Sun et al. compared MVD CD34 in laryngeal cancer (26.5 ± 6.4) with the peritumoural tissue (12.2 ± 4.0) and found a significant difference (*p* < 0.05) among them, but also confirmed a higher MVD CD34 in patients with lymph node metastasis than that in the N0 group [17]. They suggested that MVD CD34 has an important role in promoting metastases in laryngeal cancer and can serve as a prognostic marker [17]. Popov et al. reported a mean MVD with CD34 in a study group of laryngeal cancer of 14.27 ± 12.92 and confirmed a significantly higher MVD in patients with metastasis than in those without [18]. Pietruszewska et al. found a statistically significant correlation between MVD CD34 and tumour size, as well as nodal metastasis [19].

The number of papers considering the involvement of hypoxia factors in laryngeal cancer is even smaller than those evaluating angiogenesis markers. Starska et al. found a statistically significant relationship (*p* < 0.05) between HIF-1α with pTNM, tumour recurrence and overall survival in patients with laryngeal cancer [20]. There are further reports that higher levels of HIF-1α can be associated with hypoxia-mediated multi-drug resistance in patients undergoing chemotherapy via the inhibition of drug-induced apoptosis of laryngeal cancer cell, leading to a decrease in drug accumulation within tumour cells [21].

Gong et al. performed a systematic review of 28 studies evaluating the association between HIF-1α factors and survival in head and neck cancer [22]. The analysis revealed HIF-1α overexpression significantly correlated with worse survival outcomes in oral carcinoma, nasopharyngeal carcinoma and oropharynx carcinoma, but not in laryngeal cancer [22].

The above-mentioned studies analyse the influence of angiogenesis and hypoxia in invasive laryngeal cancer. The identification of biochemical factors affecting the progression of intraepithelial atypia and dysplasia in vocal fold lesions may be pivotal in the evolution of treatment methods and prevention of invasive laryngeal cancer development. The advancement of angiogenesis and the level of hypoxia in low-grade dysplasia suggest the occurrence of significant molecular changes already at play in the early stages and may influence further processes, ultimately leading to neoplastic transformation.

The possibility of novel treatment options comprising of antibody therapy that inhibit neoangiogenesis (bevacizumab) encourages the need for further research on neovascularization in intraepithelial vocal fold lesions. Such therapies, in the case of intraepithelial lesions, could be administered topically or as intralesional antibody therapy. Reoxygenation of the laryngeal epithelium at the early stage of hypertrophic pathology or undergoing low-grade dysplasia can potentially inhibit progression of dysplasia, atypia and invasion.

## Conclusions

The stage of low-grade dysplasia in intraepithelial vocal fold lesions is related to significant advancement of angiogenesis together with the highest hypoxia level. Neoangiogenesis and hypoxia during the early stages of vocal fold intraepithelial dysplasia may influence progression of cellular atypia and the further stages of carcinogenesis.

## Electronic supplementary material

Below is the link to the electronic supplementary material.

Supplementary material 1 (DOCX 14 KB)
