# Supplementary material for: Microvascular density and hypoxia-inducible factor in intraepithelial vocal fold lesions
Source: Eur Arch Otorhinolaryngol. 2019 Mar 6;276(4):1117–25. doi: 10.1007/s00405-019-05355-2 (PMC6426810; doi:10.1007/s00405-019-05355-2)
Supplement: Supplementary file 1 — Supplementary material 1 (DOCX 14 KB) [file 405_2019_5355_MOESM1_ESM.docx]

The correlation of mean CD31 MVD, mean CD34 MVD and HIF-1α expression in intraepithelial laryngeal lesions between smokers and nonsmokers.

In our study group only 7 patients were nonsmokers. The correlation with Mann-Whitney test of biomarkers’ expression between smokers and nonsmokers revealed no statistically significant differences either for the whole study group or for the patients with dysplastic lesions and invasive carcinoma. However the sample of nonsmokers is too small to make any conclusions about these data. We present results of statistical analysis in tables 5 and 6.

Table 5. Correlation of biomarkers’ expression with smoking for all patients.

|  | Number | Mean expression of CD31 | p-value | Mean expression of CD34 | p-value | Mean expression of HIF 1α | p-value |
| --- | --- | --- | --- | --- | --- | --- | --- |
| Smokers | 70 | 17,11 | 0,873 | 24,85 | 0,529 | 3,01 | 0,292 |
| Non-smokers | 7 | 16,00 |  | 27,03 |  | 2,57 |  |

Table 6. Correlation of biomarkers’ expression with smoking in patients with dysplastic lesions and invasive cancer.

|  | Number | Mean expression of CD31 | p-value | Mean expression of CD34 | p-value | Mean expression of HIF 1α | p-value |
| --- | --- | --- | --- | --- | --- | --- | --- |
| Smokers | 53 | 18,52 | 0,390 | 28,50 | 0,281 | 3,06 | 0,888 |
| Non-smokers | 4 | 19,83 |  | 34,65 |  | 3,00 |  |
